# Social talk in virtual spaces: an observational study of pragmatic communication in children with autism spectrum disorder during avatar-mediated interaction

**DOI:** 10.3389/fpsyt.2026.1859148

**Published:** 2026-07-15

**Authors:** Asmetha Jeyarani R., Radha Senthilkumar

**Affiliations:** Department of Information Technology, Madras Institute of Technology, Anna University, Chennai, India

**Keywords:** autism spectrum disorder, observational study, pragmatic social communication, social communication skills, social interaction, virtual reality

## Abstract

**Introduction:**

Immersive virtual environments offer organized social contexts for the observation of communication behaviors under standardized and interactive conditions. This study adopts a VR-based observational approach to examine pragmatic social communication behaviors exhibited during structured social scenarios in autistic children, with a specific focus on social reciprocity and context-appropriate verbal communication.

**Methods:**

The study included twenty-five children between the ages of 5 and 12 who were diagnosed with Autism Spectrum Disorder (ASD). A structured social scenario was presented by the Virtual Reality (VR) system to elicit social communication behaviors, including greeting initiation and response, attention to speakers, turn-taking, and context-appropriate verbal responses. Social communication outcomes were assessed using selected domains of the Indian Scale for Assessment of autism (ISAA). In addition, the Social Communication Questionnaire (SCQ) and a VR-based Pragmatic Social Communication Observation Checklist (VR-PSCOC) were used to assess in-scenario behaviors.

**Results:**

The post-VR analysis revealed measurable changes across the assessed pragmatic social communication domains during repeated exposure to the VR scenarios. Variations were observed in the ISAA social-communication related domains, including speech-language, communication, and social relationship and reciprocity scores (p<0.001). Repeated VR exposure was associated with higher observational scores, indicating observable changes in social communication behaviors within the structured virtual environment.

**Discussion:**

The findings indicate that the structured VR-based social scenarios provide a feasible environment for observing and characterizing social communication behaviors in children with ASD within controlled interaction settings.

## 1 Introduction

Autism Spectrum Disorder (ASD) involves persistent challenges in social communication, social interaction, and the adaptive use of language in daily situations (1). These challenges are often accompanied by stereotyped repetitive patterns of interests and/or behavior (2). Autistic children face difficulties in initiating and responding to social interactions, maintaining attention, eye contact, taking turns in conversations, topic maintenance, and employing context-appropriate verbal and non-verbal behaviors. These social communication challenges substantially impact peer relationships, classroom involvement, and daily functioning, even in children with significant structural language skills (3, 4).

Traditional intervention methods for improving social communication in children with ASD, including speech-language therapy and social skill training, generally need well-trained professionals (5, 6). It also necessitates prolonged, therapist-facilitated sessions in controlled clinical or classroom settings. However, these methods face practical constraints, such as the scarcity of trained professionals, high costs, and challenges in simulating realistic social scenarios for repeated practice (7). In addition, children with ASD may find it challenging to transfer acquired skills from therapeutic contexts to real-world social situations, which limits the functional impact of conventional interventions. These constraints underscore the necessity for alternative rehabilitation methods that are engaging, scalable, and capable of replicating authentic social environments.

Virtual reality (VR) (8) has been used as a novel tool in a variety of healthcare domains, such as mental health assessment (9), rehabilitation, neurodevelopmental research (10), surgical training (11), and behavioral intervention studies (12), over the past decade. VR has emerged as a promising technology for learning social interaction by offering immersive, interactive, and controllable environments (13). It helps children practice social communication skills in a safe and realistic environment, thereby reducing anxiety often associated with real-life social interactions (14). The multisensory and interactive characteristics of VR have demonstrated efficacy in enhancing sustained attention and motivation among children with neurodevelopmental disorders. Thereby, rendering it an appropriate medium for observing social communication skills within controlled virtual settings (15).

Recent research has investigated the application of VR environments to analyze social communication behaviors in autistic children, including conversational engagement, social responsiveness, and participation in interactive tasks (16, 17). In contrast to methods that focus on higher-order social cognition, such as emotion detection (18) or theory of mind (19), VR settings can be created to specifically target social communication behaviors directly. These encompass the initiation and response of greetings, attentiveness to conversational partners, and contextually appropriate verbal responses, which are essential elements of everyday social interaction in educational and communal settings. Furthermore, global rating scales are often employed to report improvements without conducting domain-specific analyses of core components, including social reciprocity, turn-taking, and context-appropriate language use (12). Previous VR-based interventions have reported improvements in social cognition and communication outcomes (20), there has been a lack of attention given to the examination of how specific immersive interaction features affect the underlying social communication mechanisms. (21) showed that interaction features like avatar gaze and anthropomorphic cues can change deeper psychological processes. However, the mechanism-focused analysis hasn’t been used much to look at social communication in children with ASD.

Recent evaluations and technology-enhanced studies further demonstrate the growing relevance of VR in the exploration of social communication and engagement for individuals with ASD. VR interventions have shown promise in enhancing social communication skills, such as conversational engagement, eye contact, and responsiveness in interactions, while sustaining elevated levels of participant involvement (22). A recent systematic review by (23) indicated that VR and other digital technologies effectively enhance communication-related skills, such as conversational initiation and social participation, although no singular framework comprehensively encompasses all aspects of social communication. Furthermore, deficits in joint attention persist as a major barrier in ASD, and recent VR/AR research has investigated immersive environments to facilitate shared attention, social orienting, and coordinated interaction behaviors (24). Given that shared attention is fundamental to extensive social communication skills, these results underscore the necessity of investigating initiation, responsiveness, and reciprocal engagement in organized settings. Moreover, structured environments for practice are provided by VR-based serious games, which have been found to be effective in promoting social interactions and pragmatic behaviors among adolescents with ASD (25).

The primary focus of previous VR-based studies in ASD has been on intervention-oriented social communication and social cognition training, rather than direct behavioral observation. (20) developed Virtual Reality Social Cognition Training (VR-SCT) for children with high-functioning autism. This approach utilized immersive VR interactions to enhance executive functioning, attention, social attribution, and emotion recognition. In the same manner, (26) conducted VR-based cognition training on young adults with ASD and observed enhancements in their ability to recognize emotions and understand the concepts of Theory of Mind (ToM) through interactive social scenarios.

Recent research has widened the scope of VR applications to include broader socio-cognitive processes such as social perception, empathy, Theory of Mind (ToM), and adaptive interaction frameworks. (27) constructed the Dynamic Interactive Social Cognition Training in Virtual Reality (DiSCoVR-A) paradigm for autistic individuals to help them recognize face emotions, perceive social situations, empathize, and respond in ecologically appropriate VR environments. Current adaptive VR systems have also included individualized feedback and performance-based adaptation to improve socio-cognitive growth and participant engagement (28, 29). These findings highlight the potential of immersive settings to promote higher-order social cognition and adaptive interaction processes. Despite these developments, relatively few studies have directly examined pragmatic social communication behaviors in structured VR interaction situations.

Recent review (30) of digital interventions for ASD have highlighted the importance of immersive and interactive technologies, such as VR environments, game-based systems, and digital interaction platforms, in promoting communication, collaboration, social engagement, turn-taking, and joint attention among autistic children. However, prior research has mostly focused on intervention effectiveness and communication improvement. In contrast, the current study uses a VR-based observational approach to investigate pragmatic social communication practices in organized interaction settings.

Pragmatic social communication encompasses the appropriate use of verbal and nonverbal communication behaviors in social contexts, including the use of context-appropriate language, reciprocal interaction, shared attention, initiation, responsiveness, and turn-taking. Children with ASD usually struggle with pragmatic social communication, which has an impact on their social relationships, conversational participation, and everyday interaction functioning. The current study investigated pragmatic social communication across two domains: social relationships and reciprocity, and speech-language and communication. Social reciprocity includes initiation, responsiveness, shared attention, and turn-taking, whereas speech-language and communication focus on the context-appropriate use of communication rather than language structure or vocabulary development.

Ecologically valid VR social scenarios, including a park and a birthday party, were utilized as a structured environment for observing social communication behaviors in children with ASD. Joint attention processes are conceptually related to a number of pragmatic communication behaviors that were examined in the present study, such as greeting initiation, responsiveness to social cues, shared attention, reciprocal interaction, and turn-taking. Joint attention is considered a basic social communication mechanism that involves coordinated interaction with social partners, shared attention, and responsiveness to social cues. These abilities are often impaired in autistic children. Despite the fact that joint attention was not evaluated as an independent construct in the current study, the pragmatic communication behaviors observed during VR-based interactions reflected related interactional components.

The current study is further informed by social communication and technology-mediated interaction perspectives, which suggest that structured and interactive environments might aid in the observation of socially meaningful actions through contextual involvement. Immersive learning environments, such as VR, offer controlled yet ecologically relevant settings that encourage recurrent contact, active participation, and context-based social communication experiences. These settings have been increasingly investigated in ASD research as technology-assisted settings for studying social interaction, engagement, and communication-related behaviors.

The study emphasizes the characterization of behavioral reactions and changes in observable social communication behaviors during VR-based tasks, utilizing caregiver-reported metrics and task-based observations, rather than assessing treatment efficacy. The focus is on functional and quantifiable communication behaviors to explore the potential of VR as an auxiliary observational environment for examining social communication behaviors in children with ASD. A systematic VR-based observational framework is presented for evaluating social communication in autistic children. The suggested checklist facilitates direct observation of behaviors by incorporating standardized social scenarios into immersive VR environments. Such behaviors are often difficult to capture using caregiver reports or clinical-based evaluations, making this a repeatable and non-clinical technique for detailed behavioral analysis.

### 1.1 Research questions and hypotheses

VR environments are progressively utilized to study social communication in autistic children. However, quantitative evidence regarding the emergence of communication behaviors in structured virtual social contexts is limited. This study used an exploratory, behavior-centric methodology to investigate the social communication patterns of children with ASD during VR social interaction tasks. Thus, the following Research Questions (RQ) are formulated:

RQ1: What pragmatic social communication behaviors, such as greeting initiation, turn-taking, contextual verbal responses, and attention to social cues, are displayed by autistic children during interaction with VR-based social scenarios?

RQ2: Do caregiver-reported social and communication scores and VR-based observational measures differ among autistic children across repeated VR task sessions?

Hypotheses:

H1: Children with ASD exhibit measurable social communication behaviors while participating in VR-based social interaction tasks.

H2: Caregiver-reported social communication measures and VR-based observational measures demonstrate observed variation across repeated VR interaction sessions.

The research questions were designed to define behavioral patterns during repeated VR exposure, given the exploratory observational approach of the study, and not to evaluate the effectiveness of an intervention.

## 2 Materials and methods

### 2.1 Research design

The study aims to investigate the patterns of behavior related to repeated exposure to VR-based social interaction scenarios and to define changes in social communication behaviors in autistic children. A single-group pre-post observational design was implemented. In order to characterize the baseline, quantitative data were acquired using a standardized caregiver-report scale, the Indian Scale for Assessment of Autism (ISAA), while the primary outcome measure was a task-specific VR-based observational checklist. The design was chosen to investigate the behavioral variation of participants over time in a structured, non-clinical educational environment. The study had a single-group pre-post observational design without a control condition. Thus, the approach aimed to define behavioral variation during repeated VR exposure rather than evaluate the intervention efficacy or causal linkages.

### 2.2 Participants

A total of 29 children with a prior diagnosis of ASD, established according to the Diagnostic and Statistical Manual of Mental Disorders, Fifth Edition (DSM-V) criteria, were included in this study. Participants were recruited from a rehabilitation school, and their ages ranged from 5 to 12 years. Institutional permission and written informed consent from parents or primary caregivers were obtained prior to participation. This study employed an exploratory observational design, with the sample size established based on participant availability in the rehabilitation environment and feasibility considerations, rather than a predefined statistical power analysis. Inclusion criteria included the following:

1. Existing clinical or educational records contain a documented diagnosis of ASD.
2. Ability to endure brief VR exposure under supervision.
3. Primary caregiver or close family member who is accountable for the child’s daily care and is between 23 and 59 years of age.
4. Primary caregivers were required to have adequate reading and comprehension skills to complete the caregiver-reported questionnaires, with language support provided when necessary.

The study was administered as an observational behavioral study, without any medical intervention or diagnostic decision-making. It did not include any additional clinical assessments or standardized language tests. Four children discontinued their participation during the data collection period. The final analysis encompassed data from the remaining participants. **Table 1** illustrates the key attributes of the study participants. The study comprised children with ASD ranging from mild to severe. Baseline ISAA scores show clinically relevant impairments in social communication. The caregiver-reported ISAA questionnaire was administered in either English or a translated version of the native language in Tamil, depending on the caregiver’s preference. Non-English speakers were given a translated version, with support as needed.

**Table 1:** Baseline characteristics of participants (n = 25).

Variable	Value
Age (mean ± SD)	8.3 ± 1.7 years
Age range	5–12 years
Sex (M/F)	16/9
ISAA Total Score (Median, IQR)	95 (87-103)
ASD Severity (n)	Mild: 6, Moderate: 13, Severe: 6

#### 2.2.1 Exclusion criteria

The exclusion criteria encompassed the following: (i) a history of epilepsy, seizure disorders, or medical conditions linked to photosensitivity that might elevate risk during VR exposure; (ii) significant sensory hypersensitivity, visual impairments, vestibular challenges, or intolerance to head-mounted displays that could hinder safe participation; (iii) pronounced motion sickness, or an inability to endure brief VR exposure sessions; (iv) severe behavioral dysregulation or distress that obstructed safe engagement in the VR environment; and (v) incapacity to complete the VR sessions despite familiarization and supervised assistance.

### 2.3 Study personnel and session administration

The VR sessions were administered by trained research personnel in a supervised environment. The session team was composed of two independent observers who were responsible for behavioral scoring and one facilitator who was responsible for directing the child through the VR tasks and ensuring safety. The facilitators ensured that the children were at ease with the VR device by providing standardized instructions. The supervised environment involved direct supervision by the facilitator and research observers during all VR exposures. The facilitator was present at all sessions to guide task progression, help participants adapt to the headset, check participants’ comfort, and ensure safe interaction with the VR system. Research personnel were trained in advance on session procedures, management of VR equipment, observation protocols, and identification of participant discomfort. Similar supervised VR exposure paradigms, including therapist supervision, session monitoring, and participant assistance, have been reported in prior VR ASD investigations (31).

Therapeutic training, corrective feedback, or behavioral intervention was not offered. Observers were instructed on the utilization of the social communication observation checklist prior to data collection. Observers individually evaluated recorded sessions employing established scoring standards. Video-based evaluation, item specifications, and inter-rater concordance protocols were utilized to reduce scoring variability. Complete observer blinding to session order was unachievable due to the study’s repeated observational design; still, independent scoring and agreement assessments were employed to mitigate potential scoring bias. To promote comfort and reduce tiredness, each session lasted only 20–25 minutes. Breaks were offered as needed, and participation was halted if discomfort was detected. Children were regularly observed for indicators of discomfort or adverse responses, such as apparent distress, refusal to continue, excessive fatigue, dizziness, intolerance of the headset, agitation, or withdrawal from engagement. If such responses were observed or if the child was unable to continue participation comfortably, sessions were promptly paused or terminated.

### 2.4 Instruments

The measurement framework utilized three complementary tools, each serving a specific purpose. ISAA served as a clinical reference metric to delineate baseline severity of autism related social communication and facilitate contextual interpretation. SCQ (32) was used to gather caregiver insights on the aspects of everyday social communication. The VR-PSCOC functioned as the principal observational instrument to record task-induced pragmatic behaviors during successive VR interactions. Consequently, the three measures aimed to offer contextual, caregiver-reported, and direct observational findings rather than an unbiased assessment of short-term behavioral modification.

#### 2.4.1 Clinical reference measure: indian scale for assessment of autism

**Figure 1:**
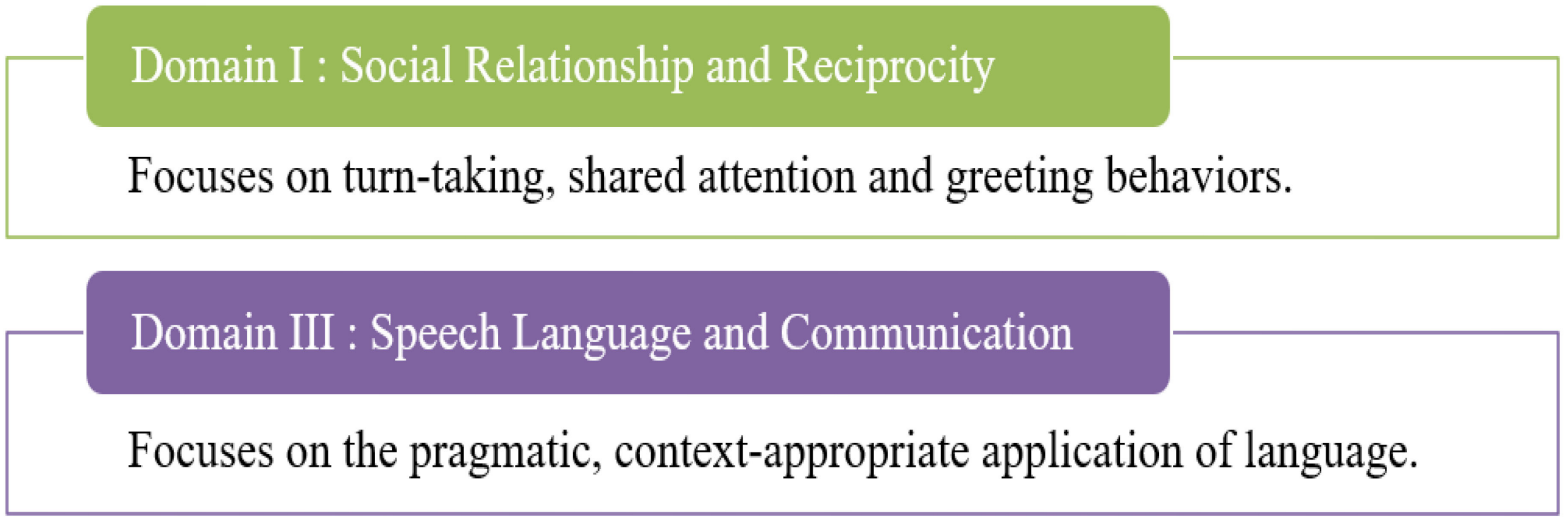
Clinical baselines based on ISAA domains.

ISAA (33) was utilized as a clinical reference tool to characterize the baseline severity of autism-related social communication among participants. ISAA is a clinician-administered evaluation that comprises 40 items across six domains. It is rated on a 5-point frequency scale (1-Rarely, 2-Sometimes, 3-Frequently, 4- Mostly, 5-Always). The assessment is not a caregiver self-report questionnaire; rather, it is derived from direct observation, structured interaction, and information provided by caregivers. However, ISAA was not employed to assess the effectiveness of the intervention or as a caregiver-reported outcome measure. Its function was restricted to (i) characterizing baseline autism severity, (ii) offering contextual clinical information, and (iii) assisting in the interpretation of observational findings based on VR. Out of five, only two ISAA domains that were pertinent to social communication were taken for consideration and are shown in **Figure 1**.

Social reciprocity describes the ability to initiate and respond properly during social interactions, such as turn-taking, shared attention, and greeting behaviors. This study does not concentrate on language acquisition or linguistic complexity despite the fact that domain III of ISAA incorporates speech and language components. Rather, it underscores the application of language, including the use of verbal responses that are appropriate for the context of typical social interactions. ISAA was not used as an intervention outcome measure to record transient behavioral changes. Variations in observed scores were utilized to promote the contextual understanding of social communication traits.

#### 2.4.2 Caregiver reported measure: social communication questionnaire

The Social Communication Questionnaire (SCQ) was employed to collect caregiver-reported information (34). The SCQ is a screening tool that is filled out by a caregiver, and it is designed to test children with ASD on their social interaction, speech, and related behaviors. It consists of 40 yes/no questions that are completed by parents or primary caregivers in accordance with the child’s current conduct. It is appropriate for non-clinical and observational research and does not necessitate clinical trial registration.

In this study, SCQ was used to obtain caregivers’ perspectives on their daily social communication behaviors. It examines the variation across repeated VR sessions and supports the interpretation of VR-based observational measures. The SCQ scores were compared and described using descriptive analysis across sessions. However, its results did not yield any diagnostic or clinical outcome claims. SCQ was not designed to identify immediate behavioral changes post-VR exposure; instead, it was incorporated to provide caregiver-based contextual insights into everyday communication traits.

#### 2.4.3 VR-based pragmatic social communication observation checklist

**Figure 2:**
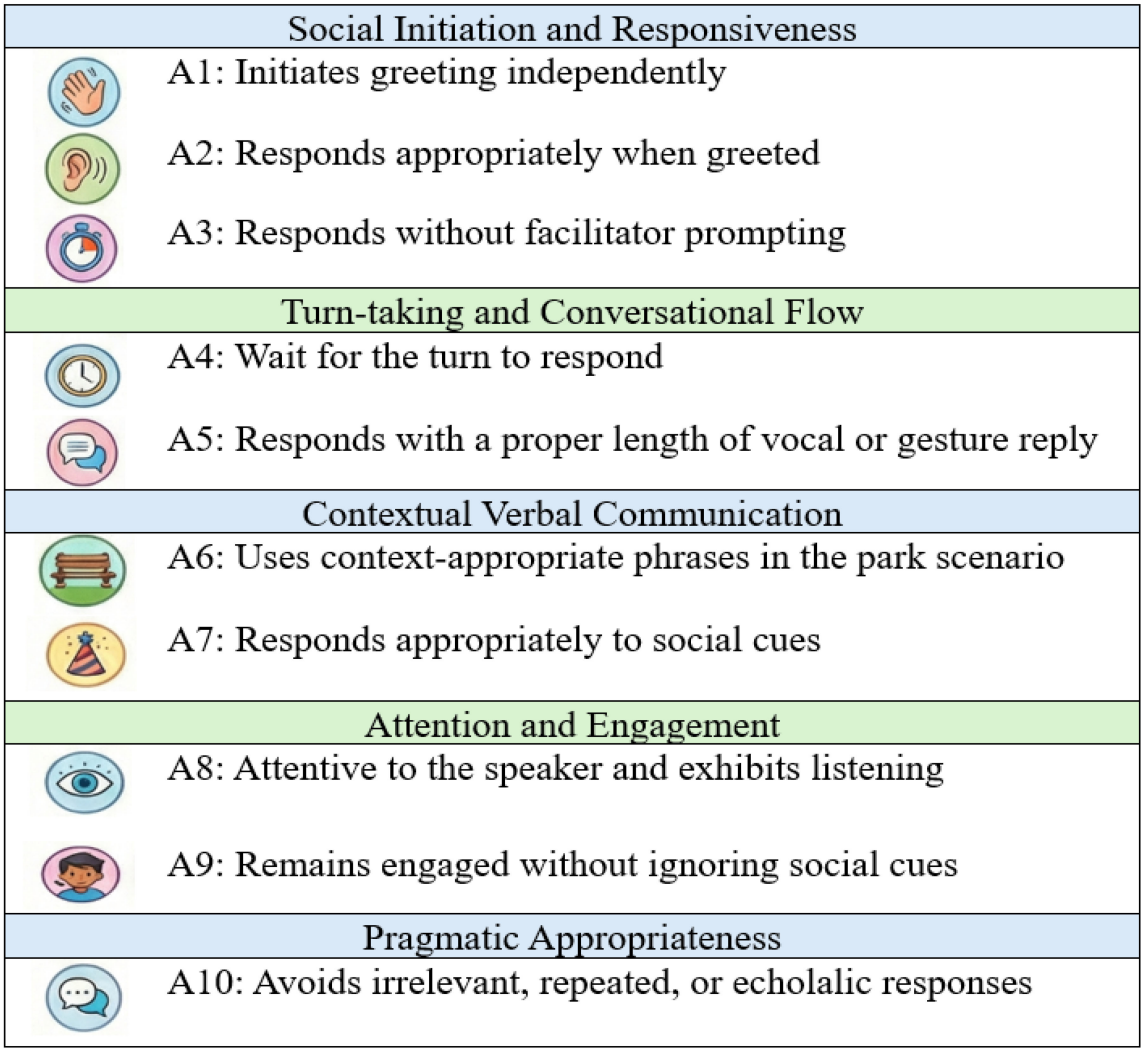
The measuring tool: VR-PSCOC behavioral items.

A VR-based Pragmatic Social Communication Observation Checklist (VR-PSCOC) was developed specifically for this study to capture task-elicited, observable behaviors during immersive VR interactions. **Figure 2** shows the measuring tool: VR-PSCOC behavioral items. The checklist included two structured VR scenarios, such as a virtual park and a virtual birthday party. The checklist was developed using domain mapping to social communication constructs such as social reciprocity and speech, language, and communication. Item selection emphasized observable, scenario-specific behaviors suitable for repeated VR-based assessment. The checklist items were conceptually mapped to ISAA domains I and III to ensure consistency. At the same time, the items were designed to be scenario-specific, directly observable, and suitable for repeated behavioral assessment during VR tasks. The checklist consists of ten predefined behavioral items, including initiating social interaction, responsiveness, turn-taking, focused attention, engagement, contextual language use, and pragmatic appropriateness.

A binary scale was employed to evaluate each item: 1 indicates that the behavior was observed independently and appropriately, without any verbal or gestural cues from the facilitator, while 0 indicates that the behavior was not observed, only after external facilitation. For scoring reasons, external facilitation referred to any verbal instruction, gesture, repetition of the prompt, or physical direction delivered by the facilitator to elicit the behavior. The total checklist score was calculated by adding item scores, yielding a range of 0 to 10. Higher scores indicate greater demonstration of social communication behaviors. Scores were calculated separately for baseline VR observation and post-observation sessions following repeated VR exposure. **Table 2** shows the description of the behavioral outline VR-PSCOC.

**Table 2:** VR-PSCOC behavioral outline for each domain.

Domain	Behavior outline
Social Initiation and Responsiveness	The child initiates and responds to social interactions with virtual characters by delivering acceptable verbal or non-verbal greetings (e.g., hi, hello). The child also responds promptly without facilitator support within 5 seconds.
Turn-taking and Conversational Flow	The child participates in reciprocal engagement with virtual avatars by waiting for their turn and responding correctly to verbal and nonverbal social cues throughout park and birthday party scenarios.
Contextual Verbal Communication	Context-appropriate language
Attention and Engagement	The child attends to social peers and remains engaged throughout the interaction.
Pragmatic Appropriateness	The child exhibits functional use of language without irrelevant or repetitive responses.

Two special educators and one speech-language pathologist with expertise in autism and social communication evaluation reviewed the content validity of the VR-PSCOC. Items were tested for clarity, relevance, and suitability for structured VR-based observation, and all items achieved satisfactory content validity (item-level Content Validity Index (CVI) ≥ 0.75). All the VR sessions were video-recorded. Two trained observers separately scored the observations. Inter-rater reliability was acceptable to high, with Cohen’s Kappa values ranging from 0.62 to 0.81, indicating substantial inter-rater agreement between raters across checklist items. Repeated exposure to identical VR settings may have enhanced familiarity with interaction patterns and must therefore be factored into the interpretation of recorded effect sizes.

#### 2.4.4 VR social communication training program

**Figure 3:**
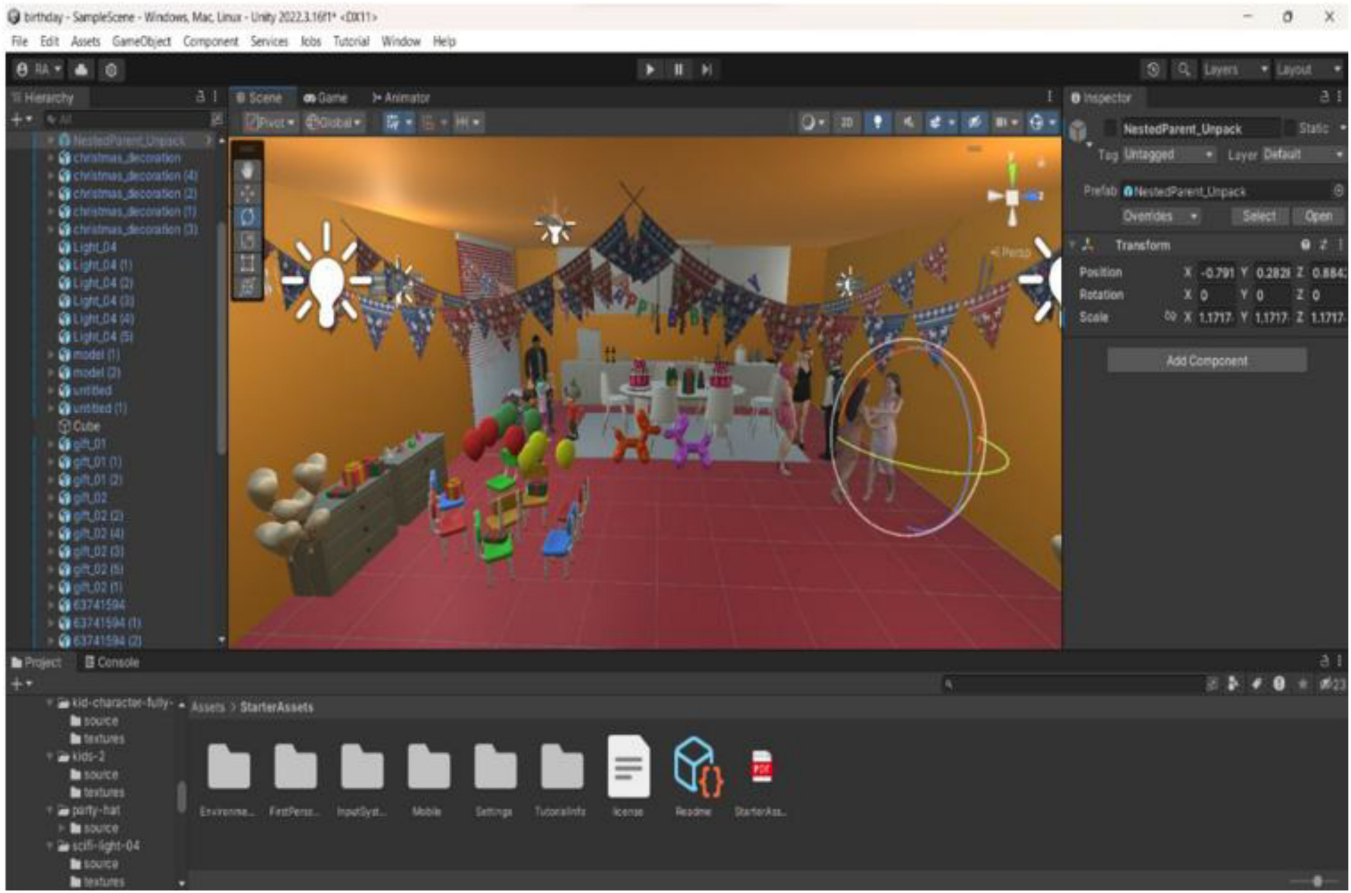
Unity interface showing the development of the VR birthday party scenario.

The VR system was developed using Unity 2022.3 LTS, while Autodesk Maya 2024 was employed to generate the 3D assets, character models, and animations. Operating under the Meta Quest runtime environment (Meta Horizon OS), the VR application was deployed on a standalone Meta Quest 3 head-mounted display. The VR headset was selected to meet appropriate ergonomic and usability requirements for short-duration exposure sessions. All sessions were conducted under direct facilitator supervision. During their VR experience, the children were constantly monitored for behavioral responses, headset tolerance, distress, fatigue, and comfort. Whenever discomfort or adverse reactions were detected, sessions were either suspended or terminated. **Figure 3** shows the unity-based design and development of the VR birthday party scenario, showing the creation of the interactive virtual environment. The system supports six degrees of freedom, enabling natural movement and interaction.

The VR scenarios were executed locally on the headgear without the need for network connectivity during the sessions. Video-based observation was employed to document participant interactions, as opposed to internal behavioral recording mechanisms. In order to guarantee consistency in data collection and behavioral observation, standardized environmental conditions, such as the session setting, supervision procedures, and observation workflow, were maintained across sessions.

**Figure 4:**
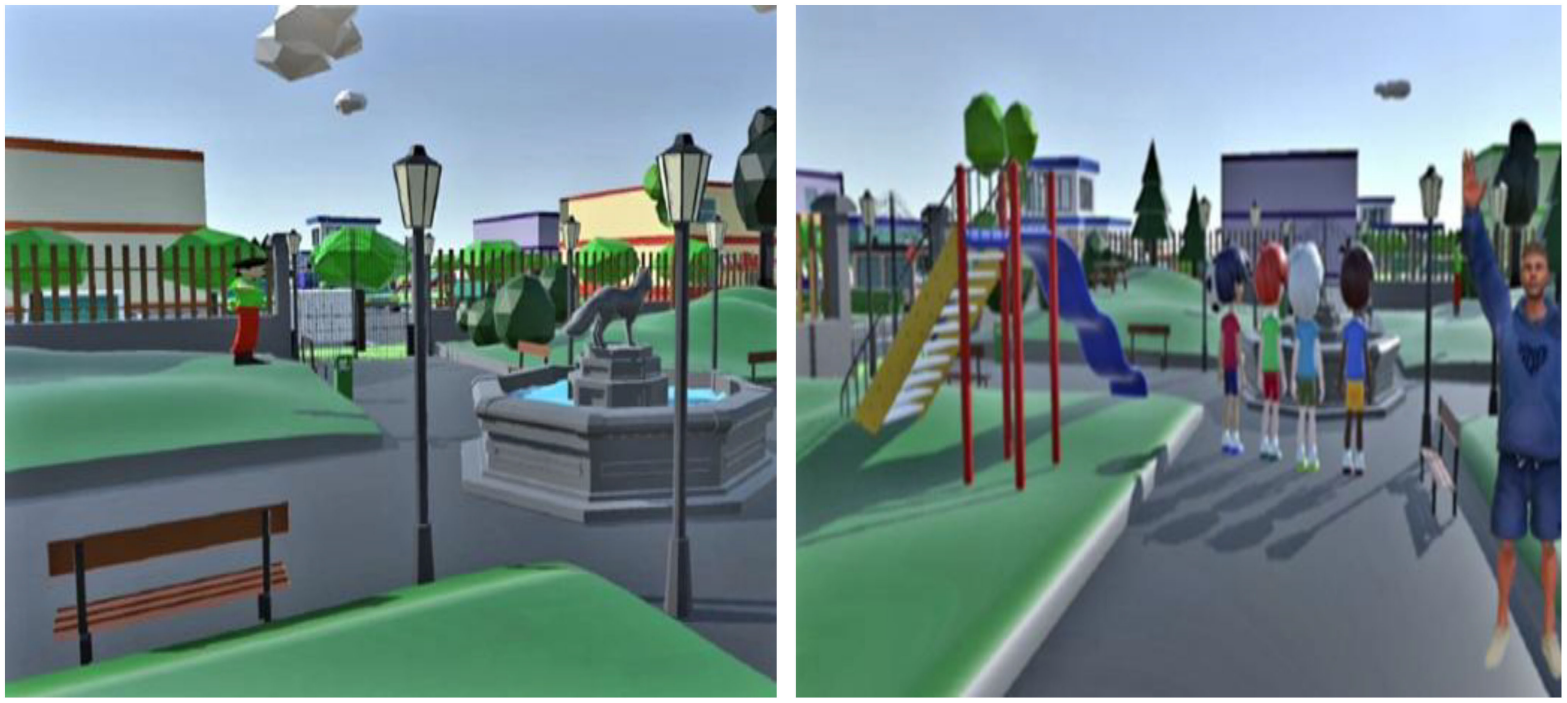
VR-based social interaction scenario designed for observational assessment (left) and VR park scenario depicting an avatar initiating a greeting (right) (17).

Two VR scenarios, such as a virtual park and a virtual birthday party, were designed to practice social communication skills. **Figure 4** displays a man waving in the virtual park scene. The virtual park scenario focused on informal interaction, greeting initiation, responsiveness, attention, and short conversations.

**Figure 5:**
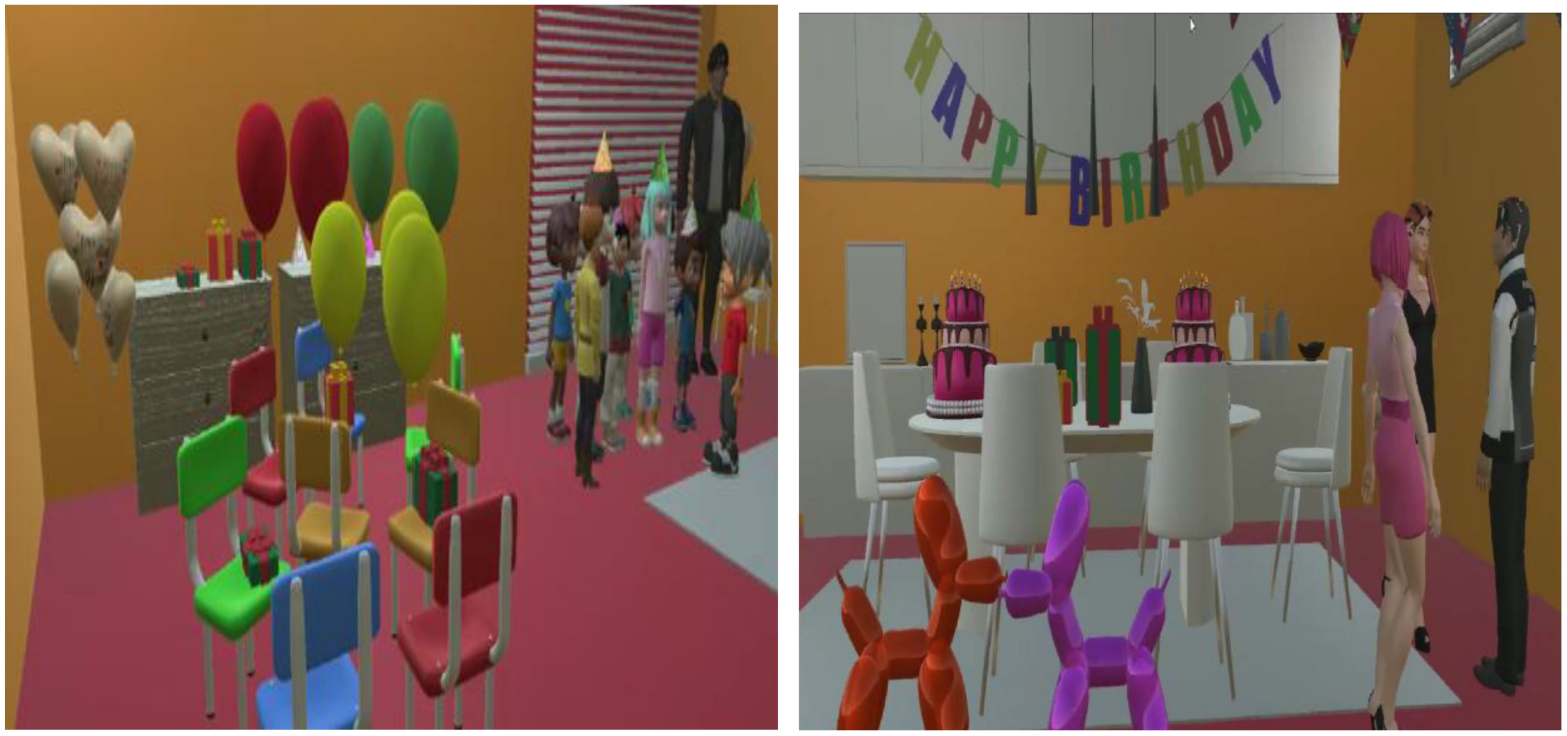
VR birthday party scene with age-matched children (left) and with adults (right).

**Figure 5** displays an example scenario of a virtual birthday party setting. The virtual birthday party scenario focused on greeting, celebratory phrases like “Happy Birthday”, group interaction, and turn-taking. Avatars used verbal and non-verbal cues, including smiling, waving, nodding, and speech prompts. All responses of the children were video recorded and observed in real time for subsequent analysis.

Each VR session adhered to a standard three-phase interaction framework, including the demonstration phase, the interaction phase, and the observation phase. The demonstration phase avatars performed brief, scripted social actions like greeting, clapping, and saying “happy birthday” to establish interaction. During the interaction phase, children engaged in free-form conversation with virtual characters and responded verbally to social cues. Children independently completed the interaction during the observation phase, and social communication behaviors were recorded using the observation checklist. The sessions were conducted in a supervised environment to guarantee the safety and comfort of all the participants.

### 2.5 Procedure

Demographic information and ISAA scores were recorded for all the participants at baseline (week 0). The children participated in the VR-based observation sessions over an eight-week period. The sessions were conducted in a rehabilitation school that was familiar to the participants and lasted between 20 and 25 minutes, all under the supervision of an adult. Participant retention was measured during the VR sessions. Four individuals dropped out during the observation period, and only those who followed the observation protocol were included in the final analysis.

Each VR session adhered to a consistent format. Initially, a brief orientation to the virtual environment was provided to the children to ensure their comfort. This was followed by direct interaction with avatars in structured social scenarios, which were aimed to encourage greetings, turn-taking, and basic conversational exchanges. During the last phase, children interacted solely with the avatars, and their social communication behaviors were observed and recorded using an observational checklist.

**Figure 6:**
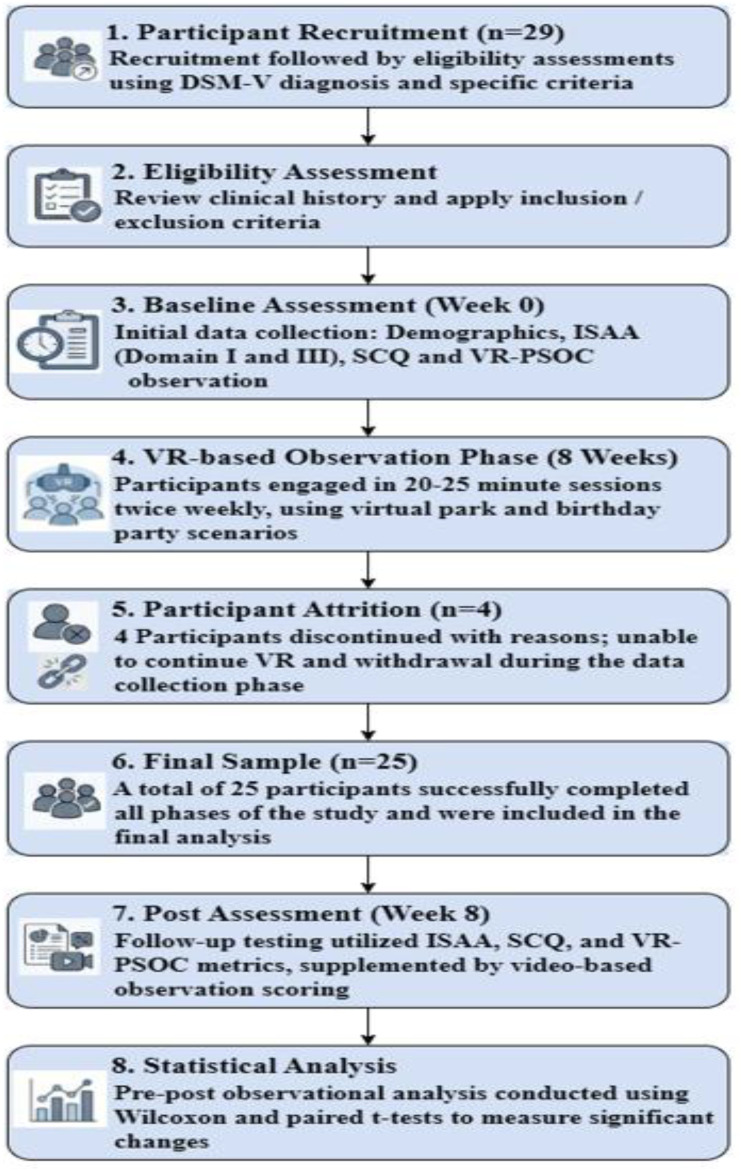
Study flow diagram of participant recruitment, assessment schedule, VR observation sessions, attrition, and analysis workflow.

Observations were made throughout the sessions to examine behavioral changes after repeated exposure to the VR scenarios. Caregiver and teacher reports were gathered to offer context for children’s everyday communication behaviors. Post-session observations were conducted using the same VR setup at the end of the observation period. The overall study procedure, including participant recruitment, baseline assessment, repeated VR observation sessions, participant attrition, and post-assessment workflow, is summarized in **Figure 6**.

### 2.6 Data analysis

Participant demographics were initially described to characterize the study sample. Descriptive statistics were used to characterize participants’ attributes, including age and sex. The ISAA social (domain I), communication (domain III), and a researcher-derived combined social-communication score (domain I + domain III), along with SCQ scores, were summarized using medians and interquartile ranges due to non-normal distributions.

The Wilcoxon signed-rank test was utilized to compare the ISAA domain scores of pre- and post-intervention ISAA. The scores from the VR-based social communication observation checklist were examined at both the aggregate and item-specific levels. This test served as the principal inferential technique for VR checklist scores. The Wilcoxon signed-rank test was the principal inferential approach due to the exploratory observational design and repeated paired measurements. Paired t-tests were performed on VR total scores following the validation of normality assumptions to offer additional examination of mean differences. Before proceeding with parametric analysis, the Shapiro-Wilk test was used to assess the normality of VR total scores. Pre-intervention VR scores demonstrated non-normal distribution (W = 0.691, p < 0.001), whereas post-intervention VR scores also showed non-normal distribution (W = 0.850, p = 0.0018). Therefore, the Wilcoxon signed-rank test was retained as the primary inferential approach due to the exploratory observational design, repeated paired measurements, and non-normal score distributions. A paired t-test was additionally performed as a supplementary analysis to examine mean differences in VR total scores, whereas the Wilcoxon signed-rank test remained the primary inferential approach because of the exploratory observational design and repeated paired measurements.

Frequencies and percentages were employed to summarize item-level behavioral performance. The statistical significance threshold was set to p<0.05. As multiple item-level comparisons were conducted for the VR-PSCOC checklist items, the results must be interpreted with caution, as formal multiple correction methods were not implemented in this exploratory observational study.

## 3 Results

### 3.1 Caregiver-reported social communication outcomes

Caregivers used the ISAA to examine the variations in children’s social communication skills across VR-based engagement sessions. **Table 3** shows the pre- and post- intervention ISAA social-communication domain scores for the 25 children who finished the VR-based observation study. The Wilcoxon signed-rank test was implemented as a result of the ordinal nature of the ISAA scale and the non-normal score distributions. Scores are presented as medians with interquartile ranges.

**Table 3:** ISAA social communication scores before and after VR intervention using the Wilcoxon signed-rank test.

Domain	Before intervention (median [IQR])	After intervention (median [IQR])	z	p
Social Relationship & Reciprocity	24 (20-27)	18 (15-21)	-4.05	<0.001
Speech-Language & Communication	25 (22-29)	19 (16-23)	-4.31	<0.001
Combined Social-Communication	49 (42-55)	37 (31-43)	-4.78	<0.001

The median score in the Social Relationship and Reciprocity domain decreased from 24 (IQR: 20-27) prior to intervention to 18 (IQR: 15-21) following interventions. This reflected variation in caregiver-reported social communication characteristics across repeated VR sessions. Similarly, the Speech-Language & Communication domain experienced a decrease from a median of 25 (IQR: 22-29) to 19 (IQR: 16-23), which reflected observable variation in communication-related characteristics across sessions. Following the intervention, the total social-communication score decreased from 49 (IQR: 42–55) at baseline to 37 (IQR: 31–43) when both domains were combined.

All observed reductions were statistically significant, with Z values ranging from -4.05 to -4.78. These findings indicate a measurable difference in caregiver-reported social reciprocity and communication characteristics observed across VR interaction sessions. Observed ISAA score variations should be interpreted as contextual observations of social communication characteristics rather than evidence of functional improvement.

The findings from caregiver-reported ISAA scores indicate a consistent reduction across both social reciprocity and communication domains following repeated exposure to VR-based interaction scenarios. Although the study used a single-group observational design, the consistent behavioral changes were observed across participants and domains after the multiple VR exposures. The lack of a control group hinders the exclusive attribution of these observed changes to VR exposure. The adoption of standardized evaluation tools enhances the dependability of the observations. Consequently, these findings are to be considered as exploratory behavioral patterns inside structured VR environments rather than as proof of causal relationships or the efficacy of interventions.

### 3.2 VR-observed pragmatic social communication performance

The VR-PSCOC measures the changes in pragmatic social communication skills during VR sessions. **Table 4** illustrates the changes in overall VR-based social communication efficacy, as assessed by the 10-item observation checklist (A1-A10), which records real-time social communication behaviors during immersive VR interactions. **Table 4a** shows the results of the Wilcoxon signed-rank test. The VR social communication score increased from 3 prior to intervention to 7 after intervention. This increase indicates observable variation in pragmatic social communication behaviors within the structured VR environment. The observed increase was statistically significant (z = -4.69, and <0.001).

**Table 4a:** VR-based social communication total scores (Wilcoxon Signed-Rank Test).

Measure	Pre (Median [IQR])	Post (Median [IQR])	z	p
VR Social Communication Score	3.0	7.0	-4.69	<0.001

**Table 4b:** VR-based social communication total scores (Paired t-test (supplementary analysis)).

Measure	Pre (Median [IQR])	Post (Median [IQR])	t	p	Cohen’s d
VR Social Communication Score	3.28 ± 0.96	7.36 ± 1.18	-13.21	<0.001	2.64

A paired t-test was also conducted to further investigate the mean differences. A paired t-test was additionally conducted as a supplementary analysis to examine mean differences; however, interpretation was based primarily on the Wilcoxon signed-rank test because normality assumptions were not satisfied. **Table 4b** shows the results of the paired t-test. The results indicated a substantial rise in mean scores from 3.28 ± 0.96 before intervention to 7.36 ± 1.18 after intervention with t(24) = -13.21 (p<0.001). The observed effect size was large (Cohen’s d=2.64); however, interpretation should take into account the repeated exposure design, structured task environment, observational characteristics of the study, the relatively small sample size, and the absence of a control group. Collectively, these data indicate that children demonstrated measurable variation in caregiver-reported and directly observed communication behaviors during VR-based social interactions.

The large effect magnitude may partially reflect repeated exposure to structured scenarios, familiarity effects, and context-specific behavioral adaptation within VR settings. The convergence of results from both non-parametric and parametric analyses supports the observed variation in communication-related behaviors within the VR environment. The considerable impact size found suggests that the magnitude of behavioral variation within the VR environment was substantial, even within the restrictions of an observational paradigm. Furthermore, the correlation between caregiver-reported outcomes and direct VR-based behavioral observations reinforces the findings’ internal consistency, implying that changes are reflected in both perceived and observed areas of social communication. Due to the study’s limited sample size and lack of a control condition, the observed effect magnitude should be regarded with caution. As a result, the data should be interpreted as exploratory behavioral observations within structured VR environments, rather than evidence of intervention effectiveness.

### 3.3 Item-wise analysis of VR-observed social communication behaviors

The VR-based social communication observation checklist items (A1-A10) were used to directly examine changes in children’s social communication behaviors throughout immersive VR social settings. **Table 5** shows the item-wise pragmatic social communication performance during VR interactions. Observable behaviors pertaining to social initiation, responsiveness, attention, contextual language use, and conversational flow were recorded by this checklist. Item-level ratings were binary and paired across pre- and post- intervention sessions. Changes were examined using the Wilcoxon signed-rank test. As indicated in **Table 5**, baseline performance was low across most items, with just 12% to 36% of children displaying adequate actions prior to intervention.

**Table 5:** Item-wise VR pragmatic social communication performance.

Item	Behavior	Pre n (%)	Post n (%)
A1	Initiates greeting independently	6 (24%)	18 (72%)
A2	Responds appropriately to a greeting	9 (36%)	21 (84%)
A3	Responds without facilitator prompting	7 (28%)	20 (80%)
A4	Wait for the turn to respond	5 (20%)	18 (72%)
A5	Responds with a proper length of vocal or gesture reply	4 (16%)	21 (84%)
A6	Uses context-appropriate phrases	6 (24%)	17 (68%)
A7	Responds appropriately to social cues	5 (20%)	15 (64%)
A8	Attentive to the speaker and exhibits listening behavior	4 (16%)	14 (56%)
A9	Remains engaged without ignoring social cues	3 (12%)	12 (48%)
A10	Avoids irrelevant, repeated, or echolalic verbal responses	5 (20%)	14 (56%)

Foundational social behaviors demonstrated the largest observable variation across repeated VR sessions. Greeting initiation (A1) increased from 24% at pre-intervention to 72% post-intervention, while appropriate response to greeting (A2) improved from 36% to 84%. Responds without facilitator prompting (A3) increased from 28% to 80%, indicating closer attention. Turn-taking behavior (A4) improved from 20% to 72%, reflecting improved conversational reciprocity. Appropriate length of vocalization or gesture (A5) showed a significant rise from 16% to 84%, indicating better regulation of expressive reactions. The use of contextually suitable terms (A6) increased from 24% to 68%, and the appropriate response to social cues (A7) escalated from 20% to 64%. In contrast, comparatively smaller observable variations were identified in sustained attention and engagement-related behaviors. Attention to speaker (A8) increased from 16% to 56%, while sustained engagement without disregarding social cues (A9) raised from 12% to 48%. Although statistically significant, these domains exhibited lower post-intervention performance in comparison to other items, indicating that attentional regulation and continuous social engagement may necessitate a more focused intervention. Avoidance of irrelevant or echolalic verbal responses (A10) increased from 20% to 56%, suggesting that there was substantial improvement in pragmatic appropriateness.

The Wilcoxon signed-rank tests revealed that all item-wise improvements were statistically significant. This suggests that there were consistent gains across multiple dimensions of communication, rather than isolated skill development. The VR-PSCOC items were binary observational measures, item-level effect estimates based on Cohen’s d were not presented. To summarize the item-wise changes, paired frequencies and inter-rater agreement values (0.63 to 0.81) are used. These values demonstrate that the observed behavioral improvements were consistently identified, as they indicate substantial to almost perfect agreement between raters.

## 4 Discussion

### 4.1 Observed pragmatic social communication behaviors during VR-based interaction

The observational study examined pragmatic social communication behaviors during repeated VR-based social interaction sessions. To identify proximal and context-specific changes, the study prioritized direct observation of pragmatic actions within structured VR settings over generalized caregiver-report measures.

The results indicate that there are observable changes in social communication during VR interactions, particularly in the areas of social initiation, responsiveness, and fundamental engagement. The VR-PSCOC captured observable behavioral variation within structured VR interactions, indicating that immersive and structured VR environments may offer structured contexts for observing pragmatic social communication behaviors. The observed behavioral changes were restricted to structured VR interaction contexts, and the transfer to naturalistic communication settings was not assessed.

The behaviors that were observed also exhibit conceptual overlap with joint attention mechanisms. Joint attention is often associated with behaviors such as greeting initiation, responsiveness to social cues, attention to speakers, shared attention, and reciprocal engagement, which involve coordinated attention processes and social interaction aspects within autism research. Hence, joint attention was not explicitly evaluated as an independent notion; several behaviors that were observed may only partially reflect interactional processes associated with joint attention in structured VR contexts.

The above pattern corresponds with known studies on autism approaches, indicating that observable behavioral changes often occur prior to changes experienced by caregivers, particularly in short-term interventions. Caregiver-report tools like the SCQ serve mainly as screening tools and depend on prolonged recall periods, rendering them less responsive to nuanced or developing changes within brief intervals (35) Thus, minimal variation in caregiver-reported metrics should be seen as an indicator of the measurement’s scope, rather than as an indication of the limitation in measurement sensitivity over short observation periods. The distinct functions of ISAA, SCQ, and VR-PSCOC should be considered when analyzing the results. ISAA offered contextual clinical characterization, SCQ represented caregiver viewpoints, whereas VR-PSCOC reported direct task-oriented behaviors during VR sessions.

The value of context-sensitive observational measures, which have been recommended for documenting short-term intervention effects in ASD research, is further emphasized by the present findings. Instruments such as the Brief Observation of Social Communication Change (BOSCC) were specifically designed to identify subtle, proximate changes in social communication during structured interactions (36). Despite the fact that the PSCOC utilized in this study is research-specific and not fully standardized, it does not adhere to the same level of psychometric standardization. It maintains a comparable conceptual focus on directly observable behaviors within controlled interactional contexts.

Comparison with other VR-based studies provides more context for these findings. It emphasizes that observable behavioral gains within structured contexts are the primary focus, rather than immediate changes in broad functional outcomes. (20) reported enhancements in emotion recognition, social attribution, and executive functioning in children with ASD after a five-week VR social cognition program. This study highlighted observable changes in social cognition-related outcomes following repeated VR exposure. Similarly, (12) identified that children who underwent VR-based rehabilitation exhibited a greater observable variation in social interaction than conventional therapy alone. Their findings reported observable differences in cognitive and social communication outcomes among children receiving virtual reality assisted rehabilitation for autistic children. These results are in accordance with the current findings, which indicate that the most significant improvements were observed in behaviors that were explicitly practiced within the VR scenarios, including greeting, responding to social cues, and maintaining engagement.

Previous VR-based studies in ASD focused mostly on effectiveness, social cognition enhancement, and communication training, rather than direct observation of pragmatic interaction behaviors. VR therapies aimed at social cognition (37) and ToM have been shown to increase emotion recognition, social perception, empathy, and adaptive interaction skills (20, 27–29). Similar recent studies on VR-assisted settings improve conversational involvement, responsiveness, and interaction quality (22, 23, 38, 39). Joint-related mechanisms, such as shared attention, responsiveness, and coordinated interaction, are also being investigated in immersive environments (31, 40, 41). In contrast, the current study focuses on pragmatic communication behaviors, such as greeting initiation, responsiveness, turn-taking, contextual language use, and reciprocal engagement inside organized VR scenarios, rather than intervention effectiveness results.

The present study varies from large-scale randomized studies like the STEPS (Social Cognitive Training Enhancing Pro-Functional Skills) trial described by (37). It targets autistic adults and focuses on psychosocial functioning and social cognition over a significantly longer intervention period with extended follow-up. In contrast, the current study uses a short-term, school-based observational design with children. It prioritizes proximal behavioral indicators within controlled VR interactions over proximal quality-of-life outcomes.

According to systematic evaluations, VR-based ASD therapies show promising but preliminary results, which are sometimes limited by small sample sizes and context-specific findings (42). Consistent with these findings, the current study used direct observational techniques to identify behavioral diversity within structured VR environments. The findings highlight the necessity of selecting outcome measures that are consistent with short-term and context-specific behavioral observations.

Given the observational design, the convergence of caregiver-reported measures and direct behavioral observations provides supportive evidence for the potential utility of VR-based environments as structured observational settings. The convergence of data from numerous evaluation methodologies improves the ecological validity and internal consistency of the results, especially in the absence of controlled experimental settings. However, the differences in session time, interaction complexity, and multi-user engagement were not investigated, which could alter observed behavioral patterns.

### 4.2 Differential effects on pragmatic social communication domains

The study’s domain-level trends show that the repeated VR exposure was related to differing behavioral patterns across pragmatic social communication domains. Observable, interaction-based categories like social initiation, responsiveness, and turn-taking showed the most significant improvements. Gains in sustained attention and continuous engagement were comparatively modest. Because of this difference, it seems that short-term VR exposure was associated with observable differences in basic behaviors, particularly those that were concrete, structured, and directly represented within immersive interaction contexts.

VR systems offer predictable ways to connect, less social pressure, and immediate contextual cues about the environment, all of which make it a rapid social routine. Earlier VR intervention studies have reported early gains in fundamental social interaction behaviors as a result of exposure to avatar-based scenarios, particularly when practice is repeated and visually grounded (20, 29). These results strengthen the view that immersive environments are particularly effective in eliciting behaviors that depend on explicit social contingencies rather than flexible inference.

Immersive VR environments encourage pragmatic social communication practices by providing predictable, visually structured, and context-controlled interaction situations. Such environments lessen social uncertainty while also providing recurrent exposure to social cues, avatar -mediated interaction, and instant contextual feedback. These features promote initiation, responsiveness, turn-taking, and reciprocal engagement by reducing interactional demands and offering structured chances for repeated practice. This interpretation is congruent with technology-mediated learning approaches in ASD, where organized and predictable environments are seen to help with social attention and communication-related behaviors.

In contrast, pragmatic domains require sustained attention, continuous engagement, and sensitivity to subtle or evolving social cues, which exhibited smaller post-intervention changes. These abilities necessitate a greater degree of attentional control, self-regulation, and the real-time integration of multimodal social information. Reviews of VR and AR interventions indicate that these systems can direct gaze and prompt attention toward avatars in the short term. However, maintaining engagement over extended interactions typically develops more gradually and often requires longer intervention duration and individualized adaptation (42). Similar patterns have been observed in school-based social communication programs, where attentional regulation and conversational maintenance emerge later than initiation or response behaviors (43, 44).

Overall, domain-level variation indicates that controlled VR environments may support the early emergence of basic interaction behaviors, whereas persistent engagement and flexible pragmatic behaviors may necessitate longer observation periods and validations in naturalistic contexts. These findings further support the utility of VR as a structured observational environment for examining communication-related behaviors in autistic children.

## 5 Limitations and future work

The present study is subject to certain limitations. Initially, the generalizability of the findings may be restricted by the comparatively small sample size. Although measurable behavioral variations were observed, the robustness of these findings across various age groups, ASD severity levels, and educational contexts should be confirmed using larger and more diverse populations.

Second, the study employed a short-term pre-post observational design that did not include a control group. Although measurable changes in social communication behaviors were observed following repeated VR exposure, the lack of a comparison group limits the capacity to determine causal relationships or the effectiveness of interventions. The repeated exposure to VR settings and the absence of rigorous observer blinding may have led to an increase in the observed effect sizes. The study utilized a single-group observational design, excluding causal inference. Thus, the observed behavioral alterations cannot be solely linked to VR exposure and should be regarded as behavioral observations within structured and controlled virtual interaction environments rather than evidence of intervention effectiveness. The present research did not evaluate the transfer of observed behaviors to real-world social contexts. Consequently, the results are confined to structured VR interaction scenarios and do not support extrapolation to real-world communication environments.

The study also did not include follow-up assessments after completion of the VR sessions. Consequently, the long-term maintenance of observed behavior variations and their generalizability beyond the structured VR environment could not be evaluated. Future studies should include longitudinal follow-up tests to examine the persistence of observed behavioral patterns and their transferability across settings.

Further constraints relate to the tolerability of VR among autistic children. Due to sensory hypersensitivity, discomfort with headset use, motion nausea, anxiety, attentional difficulties, or co-occurring conditions, not all children with ASD may tolerate head-mounted VR exposure. As a result, the study sample was biased toward participants who were able to tolerate VR exposure, which could potentially restrict the generalizability of the findings to the broader ASD population.

Additional constraints pertain to the VR system’s particular design and assessment context. The integration of these higher-order social skills into VR scenarios may be explored in future research through adaptive feedback, emotion-driven avatars, or multi-user interaction designs. The current sessions were short; future research will measure long-term usability and ergonomic impact.

Speech recognition and adaptive conversational responses were not implemented in the VR scenarios. These scenarios were based on scripted, trigger-based avatar behaviors. Future work systems could benefit from adaptive pacing and multimodal feedback to better accommodate individual learning profiles. Overall, future research should aim to extend observation duration, include controlled experimental designs, evaluate real-world transferability, and investigate the persistence of observed behavioral patterns over time. Despite these limitations, the present study offers preliminary observational evidence supporting the feasibility of VR-based environments as structured settings for examining social communication behaviors in autistic children.

## 6 Conclusion

The study illustrates that an immersive VR-based social interaction environment may support observation of measurable variation in social communication behaviors in children with ASD. In the basic domains of social relationship and reciprocity, as well as speech-language and communication, measurable behavioral changes were observed within the structured VR interaction scenarios across social communication domains. These findings indicate behavioral observations in controlled VR environments and should not be interpreted as evidence of transfer to real-world communication efficacy. Children demonstrated significant differences in key communication behaviors within VR scenarios, such as greeting initiation and response, attention to the speaker, turn-taking, and the use of context-appropriate language. Consistent behavioral patterns were detected by standardized contextual assessments and item-specific VR-based observations. These findings indicate that immersive, scenario-driven VR environments can enable observation of socially relevant communication behaviors within controlled VR scenarios. From a practical perspective, structured VR environments may benefit educational, therapeutic, and rehabilitation settings by providing controlled interaction situations for evaluating social communication behaviors in children with autism. These environments could help educators, therapists, and rehabilitation specialists assess interaction patterns, social responsiveness, and communication-related behaviors in safe and repeatable settings. In general, the results suggest that VR has the potential to be a viable observational framework for examining social communication practices for children with ASD. It is particularly useful for the purpose of analyzing communication behaviors in a controlled interaction environment.

## Data Availability

The raw data supporting the conclusions of this article will be made available by the authors, without undue reservation.
